# Supplementary material for: Inter-Species Redox Coupling by Flavin Reductases and FMN-Dependent Two-Component Monooxygenases Undertaking Nucleophilic Baeyer–Villiger Biooxygenations
Source: Microorganisms. 2022 Dec 27;11(1):71. doi: 10.3390/microorganisms11010071 (PMC9864536; doi:10.3390/microorganisms11010071)
Supplement: Supplementary file 1 [file microorganisms-11-00071-s001.zip › microorganisms-2056878-supplementary.pdf]

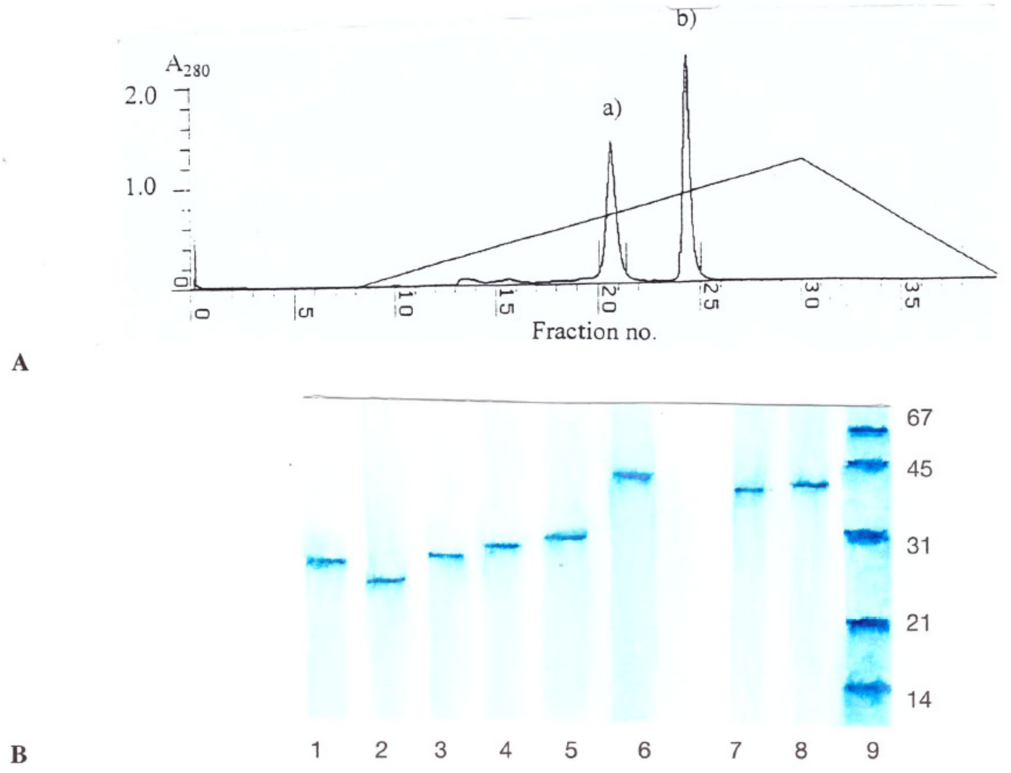


**Supplementary Figure S1A and B.** Final step purifications of both tested DKCMO isoenzymes, and all tested FRs. (A): $A_{280}$ trace of the 2,5-DKCMO (a) and 3,6-DKCMO (b) oxygenating subunits after being loaded onto an FPLC Mono-Q column and eluted with a linear gradient of 0–0.6 M KCl in 21 mM phosphate buffer pH 7.1 as the final step purification. (B): SDS-PAGE showing purification of each of the FRs.

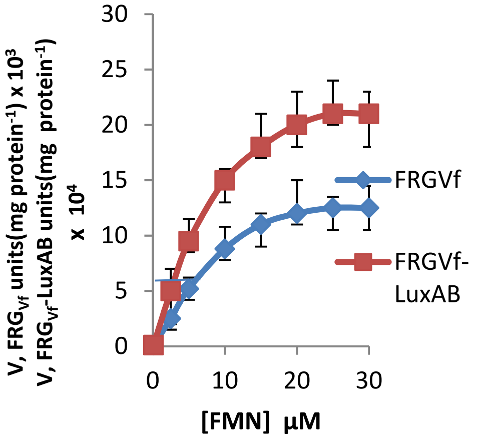


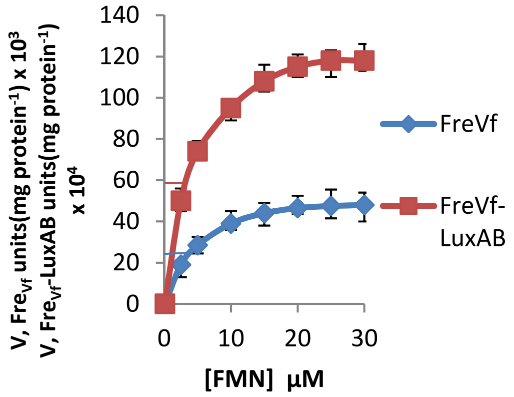


(A)

(B)

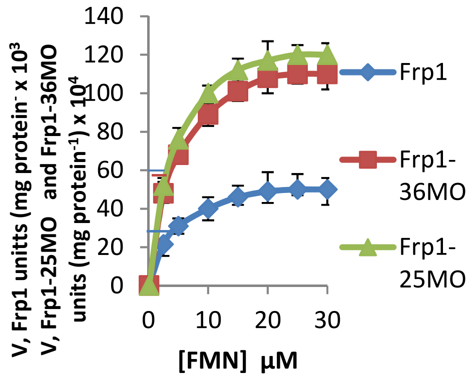


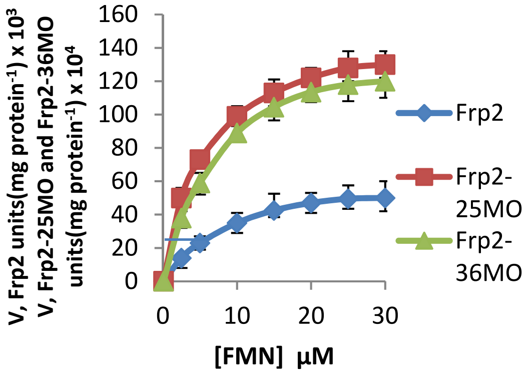


(C)

(D)

**Supplementary Figure S2A-D**. Michaelis-Menten plots of the kinetic data for the studied complementary single-enzyme and coupled -enzyme assays.

(A). Plotted data for $FRG_{Vf}$ after assay alone (single-enzyme assays) or in combination with highly purified LuxAB luciferase (coupled-enzyme assay).

(B). Plotted data for $Fre_{Vf}$ after assay alone (single-enzyme assays) or in combination with highly purified LuxAB luciferase (coupled-enzyme assay).

(C). Plotted data for Frp1 after assay alone (single-enzyme assays) or in combination with either highly purified 2,5-DKCMO or 3,6-DKCMO (coupled-enzyme assays).

(D). Plotted data for Frp2 after assay alone (single-enzyme assays) or in combination with either highly purified 2,5-DKCMO or 3,6-DKCMO (coupled-enzyme assays).

In each case the value of *v* asymptotically approached the corresponding $V_{max}$ value.
